# Tracheobronchial Foreign-Bodies in Children; A 7 Year Retrospective Study

**Published:** 2015-09

**Authors:** Soudabeh Haddadi, Shideh Marzban, Shadman Nemati, Sepideh Ranjbar kiakelayeh, Arman Parvizi, Abtin Heidarzadeh

**Affiliations:** 1 *Anesthesia Research Center, Guilan University of Medical Sciences, Rasht, Iran.*; 2 *Department of Otorhinolaryngology, Guilan University of Medical Sciences, Rasht, Iran.*; 3 *General Physician, Guilan University of Medical Sciences, Rasht, Iran.*; 4 *Department of Anesthesiology, Guilan University of Medical Sciences, Rasht, Iran.*; 5 *Community Medicine, Guilan University of Medical Sciences, Rasht, Iran.*

**Keywords:** Aspiration, Children, Foreign body, Tracheobronchial tree, Rigid bronchoscopy

## Abstract

**Introduction:**

Foreign-body aspiration is still considered one of the most important diagnostic and therapeutic issues for physicians. Mortality rates and the prevalence of diseases caused by foreign bodies in the airway are higher in children because of the relatively narrow airway and immature protective mechanisms. The aim of this study was to study the pattern of foreign-body aspiration in the tracheobronchial tree as well as the success rate of rigid bronchoscopy in children admitted to the Amir-al-Momenin Hospital, Rasht during 2007–2014.

**Materials and Methods:**

In this cross-sectional descriptive study, the required data were collected from the medical reports of all children under the age of 14 years with suspected foreign-body aspiration who were admitted and underwent explorative rigid bronchoscopy from 2007–2014. The data recorded in the checklists were analyzed using SPSS V16.

**Results:**

Out of 103 children with suspected foreign-body aspiration, a foreign body was seen in 74 children (71.8%) during bronchoscopy. Among 74 patients with a confirmed aspiration, 73% (54) were males and 27% (20) were females (P=0.68). The average age of the subjects was 34.82±33.4 months; 66.2% were aged 1–3 years. The most common complaints (symptoms) of patients were non-productive cough (48.6%), wheezing (44.3%) and respiratory distress (18.6%). The most common physical examination findings were unilateral decreased pulmonary sound (62.3%), generalized wheezing (26.1%), and crackles (17.4%). Sixty-three patients had a suspected history of foreign-body aspiration. The most frequently aspirated foreign bodies were nuts (peanuts). In total, 52.7% of foreign bodies were lodged in the right bronchial tree. In 95.9% of cases, the foreign body was completely extracted by bronchoscope. The majority of cases were admitted more than 24 hours after the occurrence of aspiration, and pneumonia was the most common complication.

**Conclusion:**

Patient history, especially initial suspicion of aspiration, coughing, wheezing and respiratory distress, can be helpful in the diagnosis of foreign-body aspiration.

## Introduction

Accidental foreign-body aspiration in the respiratory tract can lead to considerable morbidity and mortality in both adults and children. In 2000, 160 children died in the USA due to the complications induced by foreign-body aspiration, and in 2001, 17,537 children underwent treatment in emergency centers due to complications related to sudden airway obstruction (1). Foreign-body aspiration was the cause of 7% of sudden deaths among children under the age of 4 years in the USA in 1986 (2). The maximum prevalence rate is found among children below the age of 3 years (3–8). Mortality and diseases caused by airway foreign bodies are more common among children due to their narrow airway and immature protective mechanisms (9). 

Diagnosis and treatment of this condition require high awareness and an enquiring attitude to all aspiration symptoms. False or delayed diagnosis can lead to significant complications (1,3). 

Most aspirated foreign bodies are organic substances; the most prevalent being nuts and beans in children and food pieces and bone in adults. The most common inorganic bodies which are aspirated in children are beads, clips, and small parts of toys and stationery, such as the bottom of pens (4).

The signs and symptoms of foreign-body aspiration depend on the type of foreign body, its location in the respiratory tract, its size, and the length of time it remains in the tracheobronchial system (5,7). Organic substances induce more severe mucous inflammation. On the other hand, patients who aspirate small inorganic bodies tend to be asymptomatic in the long term, unless full obstruction of a terminal airway is caused (4,5). Conventionally, after aspiration, three definite clinical phases occur as follows. The first phase (initial accident) includes acute and severe coughing, choking during eating, gagging, bruising, cyanosis, and probable airway obstruction which immediately follows foreign-body aspiration. In the second phase (asymptomatic phase), the foreign body is settled and immediate simulative symptoms subside. This phase is confusing and causes delay in the patient's referral to a physician by relatives, lack of attention or diagnosis by the physician, and finally lack of suitable treatment. The third phase (complication phase) includes scar, obstruction, or infection which attracts renewed attention to the presence of the foreign body (10). In practice, choking attacks and coughing are the most prevalent clinical symptoms (6). The presence of sudden choking followed by severe coughing in a child while eating food or playing is a specific and very important indication of the probability of foreign-body aspiration. Foreign-body aspiration should be always considered in children with elongated or abnormal pulmonary symptoms (5,10). 

It has been reported that about 50% of the patients with foreign-body aspiration do not have any relevant history and 20% of children have undergone medical treatment for other diagnoses for more than 1 month before diagnosis (4).

No complications have been observed in patients who have referred to a hospital within the first 24 h after aspiration; however, if the foreign body is not removed within 24 h, it will lead to morbidity. Also, it has been demonstrated that aspiration of organic foreign bodies as well as presence of the foreign body for more than 30 days are the most important risk factors for bronchectasis(5).

Standard radiological evaluations include posterior-anterior, lateral chest X-ray and neck soft-tissue radiography; all of which should be conducted in patients with suspected foreign-body aspiration. It should be remembered, however, that the chest X-ray may appear normal during the first 24 h, and it should be noted that most foreign bodies are radiolucent (4). Radiographic findings such as atelectasis, pulmonary infiltration, and mediastinal shift may indicate aspiration.

Since no signs or clinical findings can definitively predict tracheobronchial foreign-body aspiration, investigation should be performed using bronchoscopy in suspected cases. Bronchoscopy is the best diagnostic and therapeutic method among patients with suspected foreign-body aspiration. Rigid bronchoscopy is the first option in children, because it allows for both general anesthesia and ventilation control during the procedure (2). This method limits the risk of complications, particularly if performed within the first 24 h (2,9). Morbidity resulting from bronchoscopy investigation is certainly less than that caused by an undiagnosed tracheobronchial foreign body which is removed only after a delay (11). In some studies, no complications have been observed during or after bronchoscopy (3). However, flexible bronchoscopy is preferred for the diagnosis and removal of foreign bodies in adults (4).

Bleeding, edema and laryngospasm, tracheal and bronchial spasms, and asphyxia are among the complications caused by bronchoscopy (12). 

In a study conducted in Shahrivar Hospital, Rasht, Iran from 1996–2009, the pattern of foreign-body aspiration was studied in hospitalized children; however, success of the diagnostic and therapeutic method along with probable complications was not evaluated (10). The field of foreign-body aspiration is rarely considered at the Amir-al-Momenin Educational and Therapeutic Center, Rasht, despite having a large number of referrals in the province. Therefore, attempts were made in this study to investigate foreign-body aspiration patterns in the respiratory tract as well as the success of diagnostic and therapeutic bronchoscopy among the children hospitalized in this center from 2007–2013.The results may help physicians, and even parents, in terms of early reference, diagnosis, and treatment of this disorder. 

## Materials and Methods

In this retrospective cross-sectional descriptive study, required data were collected from the files of all children (<14 years old) with suspected foreign-body aspiration in the respiratory tract who were hospitalized in the Amir-al-Momenin Hospital from 2007–2014. The required data, including the age of the patients, accompanying symptoms, the site of the foreign body in the respiratory tract, success of bronchoscopy in removing the foreign body, type of aspirated foreign body, and the presence of potential complications (such as cyanosis, tracheostomy, encephalopathy, and hypoxia, etc.), along with the cases of delayed diagnosis were gathered by the researcher from the files of all the studied patients after coordination with the hospital reception and then recorded in the designed questionnaire. However, limitations such as incomplete registry information and inaccessibility of X-ray results in the files were encountered. During different stages of the research, ethical considerations were important and the specifics of the patients were kept confidential. The variables of interest were analyzed only in the patients with a definite diagnosis of foreign-body aspiration using SPSS software, V16. 

## Results

Out of 103 children aged less than 14 years and with a probable diagnosis of airway foreign-body aspiration who underwent rigid bronchoscopy under general anesthesia by the otolaryngologist in Amir-al-Momenin Hospital during 2007–2013, 29 (28.2%) had a negative bronchoscopy, and suction and bronchoalveolar lavage were performed in the case of any secretion noticed in the airway. A foreign body was observed in 74 children (71.8%) (Table. 1), 71 of whom had their foreign body completely removed (95.5%) and received secondary evaluation. 

**Table 1:** Frequency distribution of positive and negative bronchoscopy cases in children referring to Amir-al-Momenin Hospital due to foreign-body aspiration

**Result of bronchoscopy**	**Number**	**Percent**
Negative (lack of foreign body)	29	28.2
Positive (presence of foreign body)	74	71.8
Total	103	100

The bronchoscope was taken out after ensuring lack of any foreign body and after secretions were restored. The foreign body was partially removed in two cases (2.7%), due to the fragility of the foreign body and the formation of granulation tissue; thus there was probably a need for repeated bronchoscopy in these children. In one child who was suffering from aspiration while playing with a pellet and in whom the foreign body was evident in the X-ray, the first pellet was removed by bronchoscopy; however, the second pellet was not observed during the bronchoscopy due to edema of the airway and its probable more peripheral location. Therefore, the child was referred to a thorax surgeon for the necessary treatment.

Gender distribution in the studied children and the presence or absence of foreign bodies are shown in (Table. 2). 

**Table 2:** Frequency distribution of gender in the studied children in terms of the presence or absence of a foreign body in the airways of the studied children

**Gender Group **	**Boys**		**Girls**		**Total**		**Statistical estimation **
	**Number**	**Percent**	**Number**	**Percent**	**Number**	**Percent**	
Foreign body	54	73	20	27	74	100	P=0.68
Lack of foreign body	20	69	9	31	29	100	
Total	74	71.8	29	28.2	103	100	

The mean age of the children suffering from aspiration was 34.82± 33.4 months. The youngest child was 9 months old and the oldest was 12.9 years old. Most children with aspiration were aged 1–3 years (66.2%) (Table. 3). 

**Table 3:** Frequency distribution of the age range of patients with foreign-body aspiration referring to Amir-al-Momenin Hospital

**Age (year)**	**Number**	**Percent**
Below 6 months	0	0
6-12 months	8	10.8
1 to 3 years	49	66.2
3 to 5 years	4	5.4
Above 5 years	13	17.6
Total	74	100

**Fig1:**
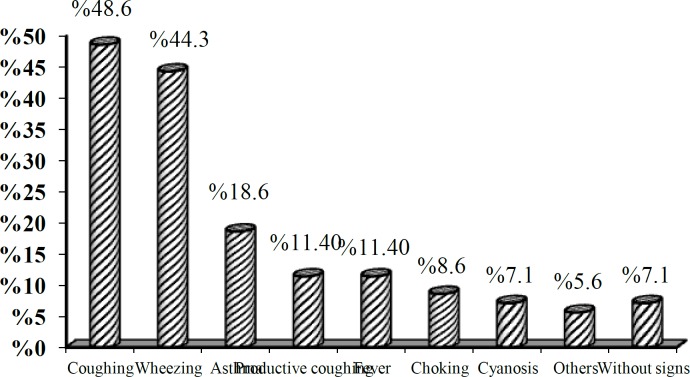
Frequency distribution of symptoms of patients with foreign-body aspiration referring to Amir-al-Momenin Hospital

Frequency distribution of symptoms in the patients with foreign-body aspiration are shown in (Fig.1). Five children were asymptomatic (7.1%), four of whom had a history of choking and sudden cyanosis followed by aspiration and one of whom was a child who was hospitalized with a diagnosis of intussusception and was suddenly found to have atelectasis during the chest X-ray studies. This child underwent bronchoscopy to rule out foreign-body aspiration.

**Fig 2:**
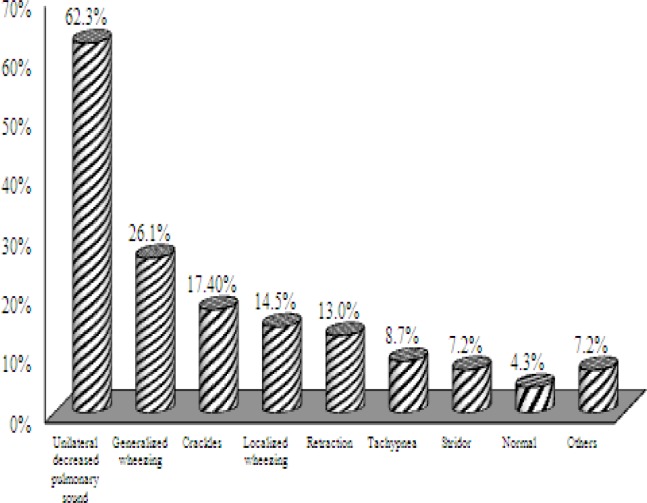
Frequency distribution of clinical examination findings of patients with foreign-body aspiration referring to Amir-al-Momenin Hospital

No information was found in five cases in terms of clinical findings; therefore, the most prevalent findings from the clinical examinations of 69 children are shown in (Fig.2). 

**Fig 3:**
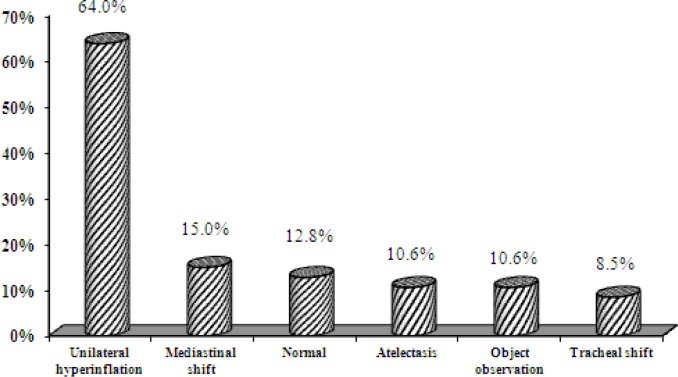
Frequency distribution of X-ray findings of the patients with foreign-body aspiration referring to Amir-al-Momenin Hospital

Among the 74 studied patients, information relating to the chest X-ray was only recorded in the files of 47 patients, as shown in (Fig.3). 

Information relating to the nature of the aspiration (by parents or scene witnesses) was available in 68 files, among which 63 children (92.6%) had a history of suspicion of foreign-body aspiration.

Information relating to time to referral was collected in 68 out of 74 children due to incomplete files. Time to referral in most of these children was longer than 24 h (75%), while only 17 cases referred before 24 h. According to the information relating to 65 files, 15 children (23.1%) had a final diagnosis of pneumonia and asthma and initially received medication for these indications. Eleven of these 15 children (73%) had a history of suspected aspiration.

**Fig 4:**
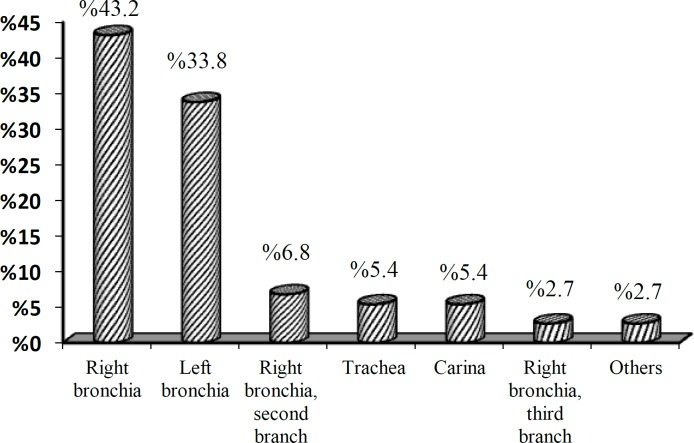
Frequency distribution of foreign-body location in patients with foreign-body aspiration referring to Amir-al-Momenin Hospital

The frequency distribution of aspirated foreign-body type is shown in Table 4. Frequency distribution of the location of the aspirated foreign body in patients is shown in (Fig.4). 

Forty-five of 74 patients (60.8%) referred to the center with complications caused by foreign-body aspiration (pneumonia 82.2%, atelectasis 9%, cyanosis and hypoxia 4.4%, apnea and need for intubation 2.2%, and severity of asthma attack 2.2%). No complication was found in 24 patients on arrival (32.4%). Forty-one of 45 patients with aspiration complications (91.1%) referred after 24 hours of aspiration; two (4.45%) within the first 24 h, while the referral time of the two other patients is not recorded in the file.

The condition of the patients after bronchoscopy and removal of the aspirated foreign body was as follows: 68 children (91.9%) were conscious and stable, six children needed intensive care (one child due to sleepiness and sinuous arrhythmia, two due to airway edema and the resulting respiratory distress, one due to sleepiness and hypoxia, and one due to pneumonia and elongated aspiration). The last case was a 35-month-old child with a history of neuromuscular disease who had a tracheostomy tube and was referred with respiratory distress and cyanosis due to the breakage of a part of the metal tracheostomy tube and fall in the trachea. 

## Discussion

Foreign-body aspiration in the airway is one cause of respiratory problems in children, leading to an increased risk of mortality, and is a prevalent cause of medical emergencies (13). The maximum risk of foreign-body aspiration in the airway of children occurs during the first 3 years of life and often in the 1–3-year age range (3,4,5,8,10,11). In the present study, 66.2% of the cases of aspiration occurred within the age range of 1–3 years. The tendency of children at this age to put objects in their mouth, combined with the immature neural mechanisms which coordinate aspiration and respiration, and the habit of playing and laughing at the time of eating make children susceptible to aspiration (8). In this study, most (74.3%) of the aspirated foreign bodies were organic substances such as nuts or sunflower seeds. This finding is consistent with the results of other studies (2,3,5,9,11,13). In the study by Farzizadeh, sunflower seed was the most prevalent aspirated foreign body (10). Nuts and vegetable grains were the most common aspirated bodies.

In many studies, the incidence of foreign-body aspiration has been reported to be higher in boys than girls, and it has been suggested that this may result from differences in their activities (playfulness) (3,5,8,11,13). The study by Farzizadeh is contrary to this trend, reporting an incidence of aspiration that was identical in both genders (10). In the present work, the incidence of foreign-body aspiration was higher in boys than in girls; however, there was no statistically significant relationship between gender and the presence or absence of a foreign body in the airways of the children studied P=0.68).

In this study, non-productive coughs (48.6%), wheezing (44.3%), and respiratory disorders (18.6%) were the most prevalent complaints among the patients. Unilateral decreased pulmonary sound was the most prevalent clinical finding and the existence of a history of suspected foreign-body aspiration (observation of aspiration by others or acute choking following foreign-body aspiration) had high prevalence (92.6%). 

Farzizadeh et al. found that coughing and respiratory disorders were the most prevalent complaints of patients. A triad of foreign-body aspiration (initial suspicion, respiratory distress, and choking attack during eating) had a high prevalence and it was noted that history, particularly initial suspicion of foreign-body aspiration and choking while eating, can be helpful in diagnosis, considering their high prevalence (10). In the study by Arji et al., choking and observation of aspiration by others had maximum sensitivity and specificity among the clinical findings (14).

In the work conducted by Thomaskea et al. on the diagnostic value of clinical findings and symptoms, unilateral decreased pulmonary sound and unilateral emphysema in the chest X-ray were the most sensitive (53–79%) and specific (68–88%) findings in both of the studied groups. Furthermore, it was demonstrated that the clinical triad (coughing or sudden choking, wheezing, and unilateral decreased pulmonary sound) had high sensitivity, while a history of coughing, sudden choking, or aspiration observed by others were the most important criteria in the correct diagnosis of foreign-body aspiration. In contrast, a positive history of coughing or acute choking in children with symptoms less than 2 weeks in duration or a history of continual coughs in children with symptoms longer than 2 weeks in duration showed low specificity (8–16%) (11). In the present study, unilateral decreased pulmonary sound was the most prevalent clinical finding; however, the most prevalent complaints were coughing, wheezing, and respiratory disorder.

In the work by Reina, presence of an appropriate history, clinical examinations, and analysis of diagnostic tests were reported to have high importance in any condition (7). In another study, coughing and cyanosis were the most prevalent complaints, decreased pulmonary sound and localized and generalized wheezing were the most prevalent clinical findings, and 15% of the patients did not have abnormal clinical findings (13).

In the study by Ghafari et al., topical emphysema (44%) and atelectasis (16%) were the most prevalent radiological findings. Also, the most important reason for delayed diagnosis was absence of symptoms at the acute stage, normal clinical examination, and normal chest X-ray (13). In the present study, the most prevalent radiological finding was unilateral emphysema (64%), which is consistent with the results of Ghafari et al.

In the study by Thomas et al., the sensitivity and specificity of unilateral emphysema were reported as 53.1–60.2% and 82.5–87.7%, in the chest X-ray respectively (11). In the present study, unilateral emphysema and mediastinal shift were the most prevalent findings in the chest X-ray; indeed, chest X-ray was normal in 10.2% of cases. 

The rate of normal chest X-rays in the different studies was reported to be between 19 and 25% (5,6,10). In the study by Mansourian et al., the sensitivity, specificity, positive and negative predicting values, and precision of the chest X-ray at the time of exhalation were 65%, 50%, 85%, 25%, and 62.5%, respectively (15). Therefore, a normal chest X-ray does not always preclude occurrence of aspiration in patients with a history of aspiration and the presence of clinical findings (15). This finding is in line with the present results.

Thomaskea showed that the type of aspirated foreign body and the length of time the foreign body remains in the respiratory ducts are the most important factors relating to complications. Due to the severe inflammatory reactivity of the tracheobronchial tree to organic bodies, fever, bronchopneumonia, and other complications occur more rapidly with organic rather than inorganic aspiration. In the situation when a patient undergoes treatment for the removal of a foreign body within the first 24 h, almost no complications will be caused (11).

Delay in the diagnosis of foreign-body aspiration of longer than 4 weeks in the study by Malek et al. led to a considerable incidence of complications such as pneumonia, bronchectasis, and tracheoeso- phageal fistula (8). 

In the present study, the most prevalent complication observed was pneumonia, with other complications including atelectasis, hypoxia, cyanosis, and severe asthma attack. It is necessary to note that most patients with complications (91.1%) in the present study referred 24 h after aspiration. 

Late or delayed manifestations can result from the false diagnosis of physicians. Negligence of parents in referring to therapeutic centers, delayed referral by hospitals or clinics, and absence of family members at the time of sudden choking can all lead to a delayed diagnosis. Increased general awareness and training of physicians can help prevent complications (8). In the study by Fakhim, 84 patients with an initial diagnosis of pneumonia and 22 patients with an initial diagnosis of asthma underwent treatment. The mean time to hospital referral was 24 days, and only 24.5% of the patients referred within the first 24 h of aspiration. In this study, atelectasis was found in 21% of the delayed cases. While examining the reasons for delay in the referral of a patient, the inattention of parents (58.9%) and the false diagnosis of physicians (41.1%) were found to be important factors in the delay in diagnosis (9). 

In present study, only 25% of the children referred within the first 24 h. fifteen children had already undergone treatment with other diagnoses, and there was a suspected history of aspiration in 11 cases. 

In Jose's study, 25% of the foreign-body aspiration was not diagnosed in the first visit, despite the existence of typical symptoms after acute choking (6). 

In the present study, foreign bodies were more commonly located in the right bronchia, which was similar to other works (3,6,9); however, some studies have reported the observation of foreign bodies in the left bronchia (2,8). In the study by Farzizadeh et al., the location ratio of foreign bodies in the right and left bronchia was equal in children under 1 year of age and higher in the right bronchia at other ages (10). In another study, there was no difference between the right and left bronchia (13). 

In the present study, negative bronchoscopy (no observation of foreign body) was 28.2%. Negative bronchoscopy in the study by Jose et al. was below 10%. The outcome of a negative bronchoscopy was better than leaving the foreign body in the bronchial tree (6). In the work by Malek et al., the negative bronchoscopy rate was 14.2%, and it was noted that a number of negative bronchoscopies is required to ensure that no foreign body is neglected (8). Therefore, if physicians doubt whether or not tracheobronchial foreign-body aspiration has occurred, they should investigate using bronchoscopy because morbidity resulting from bronchoscopy is certainly less than that caused by lack of diagnosis of a foreign body in the tracheobronchial tract (5). 

In this study in 71 children (95.9%), a foreign body was completely removed using bronchoscopy. The foreign body was partially removed in two other cases due to the fragility of the aspirated foreign body and the formation of granulation tissue, and one case was referred to a surgeon due to peripheral aspiration. Therefore, the type, duration, and location of the foreign body can play a role in the success of foreign-body removal using bronchoscopy. 

## Conclusion

Based on the present study, it is useful to consider the history, particularly the initial suspicion of aspiration, coughing, wheezing, and respiratory disorder, for the diagnosis of these symptoms. The best method of preventing serious hazards induced by foreign-body aspiration is prevention and attention to the history of patients. 
